# Perforated mixed carcinoid-adenocarcinoma in transverse colon and at gastroenterostomy site: case report

**DOI:** 10.1186/1477-7819-8-110

**Published:** 2010-12-22

**Authors:** Enver İhtiyar, Özgül Paşaoğlu, Serdar Erkasap, Barış R Karakaş, Fatih N Yaşar

**Affiliations:** 1 Department of General Surgery, Eskişehir Osmangazi University, School of Medicine 26480, Eskişehir, Turkey; 2 Department of Pathology, Eskişehir Osmangazi University, School of Medicine 26480, Eskişehir, Turkey

## Abstract

Goblet cell carcinoid of the large intestine is a rare neoplasm, usually located in ascending colon and rectum. A 60-year-old male patient underwent surgery after the diagnosis of acute abdomen. Exploratory laparotomy revealed perforation with a diameter of 1 cm at the site of the previously performed gastroenterostomy and dilatation of the right colic flexure, secondary to a solid obstructive mass located in the mid-portion of transverse colon. Histopathological investigation of the biopsies, taken from the gastroenterostomy site and the tumor, revealed mixed carcinoid-adenocarcinoma with carcinoid component, predominantly composed of goblet cells. Three cycles of FOLFOX-4 protocol was administered. Following respiratory distress secondary to pulmonary metastasis, the patient's condition deteriorated and subsequently died in the fourth postoperative month. Our aim with this paper is to point out that more cases should be reported for more effective diagnosis, histopathological study, clinical investigation, treatment and prognosis of this specific neoplasm.

## Background

Goblet cell carcinoid (GCC) of the large intestine is a rare neoplasm, usually located in ascending colon and rectum. Histologically, it is similar to goblet cell carcinoid of the appendix [1]. GCC has both endocrine and glandular differentiation. Dual differentiation probably arises from a pluripotent intestinal stem cell instead of two different mature cells. The mean age for diagnosing GCC of the appendix is 58.89 years with equal representation in both genders. Regional and systemic metastasis is common at initial diagnosis. These tumors perform aggressive behavior with tendency for metastasis and wide local dissemination [2]. Lesions are treated according to the same conventional oncologic approach to adenocarcinoma [3]. We present here, a 60 year-old male patient, who diagnose as mixed carcinoid-adenocarcinoma located in transverse colon and at gastroenterostomy site.

## Case

A 60 year-old male patient presented with complains of nausea, vomiting, abdominal distension, and no discharge for three days. He also had intermittent cramping abdominal pain, mainly located in the upper left abdominal quadrant. He had a history of prior gastric surgery, performed 26 years ago, for peptic ulcer disease. His vital signs included temperature of 36.4°C, blood pressure of 100/80 mmHg, pulse rate of 60 beats/min, respiratory rate of 22 breaths/min. On physical examination, the scar of the midline incision was inspected and the abdomen was distended and tender to palpation with guarding. Routine hematological and biochemical investigations were within normal limits except for raised total leucocytes count (32,000/mm³). Serum carcinoembryonic antigen (CEA) and cancer antigen (CA) 19-9 levels were not elevated on the postoperative 3rd day of the follow-up. Plain X-ray of abdomen revealed few fluid levels and free gas in subphrenic spaces whereas the abdominal ultrasonography showed no finding but diffuse intestinal gas. The patient underwent surgery after the diagnosis of acute abdomen was made. Exploratory laparotomy revealed perforation with a diameter of 1 cm at the site of the previously performed gastroenterostomy and dilatation of the right colic flexure, secondary to a solid obstructive mass located in the mid-portion of transverse colon. There were no metastatic liver lesions whereas metastatic lymph nodes were detected in mesocolon. The gastroenterostomy was reconstructed after anastomosis and the mid segment of the transverse colon with approximately 5-6 cm margins on either side of the tumor was resected.

**Figure 1:**
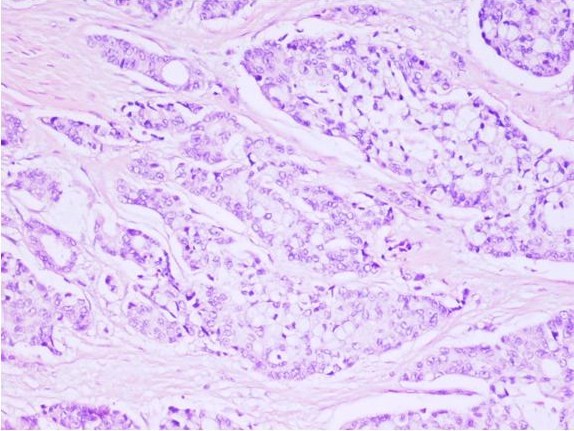
**Carcinoid component of mixed carcinoid-adenocarcinoma is composed mainly of goblet cells**. (H.E.; × 200).

Histopathological investigation of the biopsies, taken from the gastroenterostomy site and the tumor, revealed mixed carcinoid-adenocarcinoma with carcinoid component, predominantly composed of goblet cells. Ulcero-vegetative mass in the transverse colon with the size of 5 × 5 × 1.5 cm, infiltrating the intestinal serosa, and three tissue samples, each measuring approximately 2.5 × 1.5 × 0.3 cm, taken from the gastroenterostomy site were microscopically similar and had the characteristics of mixed carcinoid-adenocarcinoma with carcinoid component, predominantly composed of goblet cells (Figure 1). Tumor invasion in all layers of the transverse colon and the gastroenterostomy site are accompanied by perforation. Immunohistochemical stains showed that neoplastic cells were positive for neuron-specific enolase (NSE), synaptophysin and E-cadherin and negative for chromogranin. Ten metastatic lymph nodes were detected in mesocolon. At three months postoperatively the needle biopsy specimen of the liver revealed metastasis.

The 24 hours urine vanillylmandelic acid (VMA) level was within normal range on the postoperative 5th week of the follow-up. In-111 octreotide scintigraphy detected increased uptake in the region of the para-aortic lymph node, compatible with a lesion which had the expression of somatostatin receptors. A bone scan was performed using 20 mCi of Tc-99 m MDP, and uncovered no evidence of abnormality. Three cycles of FOLFOX-4 protocol was administered by the medical oncology department. He was hospitalized three months after the operation because of poor health status. Ultrasonography of the liver showed an inhomogeneous echo texture, hyperechoic nodules with peripheral hypoechoic halos and the largest lesion with size of 3 × 2.8 × 2.4 cm was localized in the anterosuperior portion of the right lobe. A needle biopsy of the liver was positive for metastasis of the carcinoma. Following respiratory distress secondary to pulmonary metastasis, his health situation got worse and subsequently died in the fourth postoperative month. In additionally, the patient was questioned about any symptoms of the carcinoid syndrome, which includes flushing, diarrhea, wheezing etc. pre-operatively once the post op diagnosis was made and post-operatively. He did not encounter any symptoms of the carcinoid syndrome.

## Discussion

Since more than 30 years ago, a new variant type of epithelial tumor of appendix has been recognized. This tumor is reported under different names including goblet cell carcinoid (GCC), adenocarcinoid, mucinous carcinoid, intermediate type of carcinoid, crypt cell carcinoma, amphicrine (endo-exocrine) neoplasia, composite tumor and microglandular carcinoma. All names except GCC have been omitted from the current World Health Organization (WHO) classification [2]. Subbuswamy et al described the first report of GCC in 1974 [4]. The histology and biology appears to be intermediate between carcinoid tumors and adenocarcinomas. This tumor appears to combine features of epithelial and carcinoid neoplasms and in addition the surface mucosal epithelium is not neoplastic. Histopathological features such as increased number of Paneth cells, increased amount of mucin secretion and presence of pancreatic polypeptide may predict a more aggressive behavior [2].

Mucocarcinoids also called mucinous or adenocarcinoids, show a quite different histological appearance from carcinoids and endocrine cell carcinomas. The tumor is composed predominantly of small clumps, strands, or glandular collections of mucin-producing cells looking like goblet cells or signet-ring cells, and intermingled with endocrine cells in a variable number and occasionally with Paneth's cells. The admixed endocrine cells comprise a variety of cell types, such as somatostatin-containing D cells, serotonin-containing endocrine cells and enterochromaffin-like cells containing histamine. They are often sparse and, in about 10% of cases, difficult to find. The tumor was originally considered to be a variant of a carcinoid. The frequent paucity of endocrine cells and more aggressive clinical nature are not consistent with such speculation. Mucocarcinoids are a variant of adenocarcinomas showing differentiation to both mucin-producing cells and endocrine cells. They occur most frequently in the appendix, but rarely in stomach. Ito et al reported only one case treated in 10 years period [5].

Even if there are many questions about histogenesis of tumors with mixed differentiation, it is hypothesized that these neoplastic lesions may probably arise from a single pluripotent stem cell as well as different mature cells [6]. Histologically, these tumors are divided into three subtypes: mixed (composite) tumors, collision tumors and amphicrine tumors. In mixed tumors, the two elements typically merge and intermingle, and in some areas transitions can be seen, such that the two components can be difficult to distinguish. A carcinoid component should compose at least one third of the tumor cell population in a composite tumor. In collision tumors, the two elements should be in intimate contact without intermixture of individual cell types. Amphicrine tumors differ from the above tumor types in that endocrine and nonendocrine epithelial cell constituents are present within the same cell [7]. These tumors behave more like adenocarcinomas than carcinoids. Two cases with mixed carcinoid-adenocarcinoma, for the first time, were reported by Moyana et al in 1988 [8].

## Conclusions

This case with the characteristics of mixed carcinoid-adenocarcinoma with carcinoid component, predominantly composed of goblet cells, is reported because of its rarity and points out that more data should be collected to develop our knowledge about diagnosis, histopathological and clinical features, prognosis, and conventional treatment of this neoplasm.

## Consent

Written informed consent was obtained from the family of the deceased patient for publication of this case report and accompanying images. A copy of the written consent is available for review by the Editor-in-Chief of this journal.

## Competing interests

The authors declare that they have no competing interests.

## Authors' contributions

Eİ performed the operation and helped manuscript preparation particularly in describe the findings and the follow-up, revised and edited most of the manuscript. ÖP performed the histopathological and immunohistochemical analyses of all surgical specimens, provided the figure of the microscopic appearance of the tumor, helped literature search, and corrected the final draft. SE helped with the editing of the manuscript and literature search. BRK and NFY were involved in preparation of initial draft and literature search. All authors read and approved the final manuscript for publication.
